# Lecture 2—What Is a Formulary, Anyway? (Or the Cliff Notes Version of Drug Stewardship and Expense Control)

**DOI:** 10.3390/pharmacy6030069

**Published:** 2018-07-20

**Authors:** Richard H. Parrish

**Affiliations:** 1 Department of Pharmacy Services, St. Christopher’s Hospital for Children, 160 East Erie Avenue, Philadelphia, PA 19134, USA; richardhenryparrish2@gmail.com; Tel.:+1-215-427-5317; 2 School of Pharmacy, Virginia Commonwealth University, Richmond, VA 23298, USA

**Keywords:** formulary, clinical pharmacy, P&T committee, rational drug therapy

## Abstract

A formulary is the product of an evaluative process, the formulary system, conducted by an expert panel that both sanctions and guides the selection, prescription, administration, and monitoring of pharmaceuticals and related items for a given environment. An expert panel, often called the Pharmacy and Therapeutics Committee (P&T), is a group of pharmacists, physicians, nurses, and administrators assembled for the purpose of providing guidance and validation for pharmaceutical utilization in a given organization. Expert panels became prominent because of qualitative and quantitative changes in drug production and marketing strategies employed by industry, the expansion of public health sector medicine, and organized, macro-level drug control systems. It could be argued that, as a clinical instrument, the formulary is predicated on the role pharmacy is perceived to play in actual direct patient care. In this lecture, the concept and defining characteristics of a formulary (the perspective of an expert panel, differences in the environment of application, and interprofessional relationships), the modifiers used to describe a formulary, which modifiers enhance or distract from its meaning, and the outlook for comprehensive and objective evaluation through the formulary mechanism are discussed.

## 1. Introduction

A formulary is the product of an evaluative process, the formulary system, conducted by an expert panel that both sanctions and guides the selection, prescription, administration, and monitoring of pharmaceuticals and related items for a given environment [1,2,3,4]. The meaning of a formulary is dependent on the perspectives of the expert panel, the environment where it is applied, and the clinical and interpersonal relationships among pharmacy, medicine, nursing, and administrative personnel [5]. Historically, formularies standardized nomenclature and recipes for naturally occurring substances and combinations [6] with little, if any, governmental influence. Today, formularies represent a sub-set of the market of mass-produced pharmaceutical products approved by the U.S. Food and Drug Administration (U.S.-FDA) and similar regulatory bodies in other jurisdictions. Formulary systems deal with issues regarding generic substitution, therapeutic interchange, and disease management. Currently, patients not only have their prescription drugs selected and procured for them by informed intermediaries; third party payers often pay the price of pharmaceuticals. Because of the degree of regulation by state and federal governments, the pharmaceutical market is not a normal market [7]. Formularies attempt to address two essential elements found in a normal market: (1) relational therapeutic and economic value calculations by those who demand or pay for pharmaceutical products; and (2) price competition and interchangeability among suppliers through some sort of contract bidding process. In this lecture, I will discuss the concept and defining characteristics of a formulary (the perspective of an expert panel, differences in the environment of application, and interprofessional relationships), the modifiers used to describe a formulary (open, closed, etc.), which modifiers enhance or distract from the provision of rational drug therapy, and the outlook for comprehensive and objective evaluation through the formulary mechanism. In addition, I will provide a brief description of the evolution of the formulary into the digital age.

## 2. The Perspective of the Expert Panel

An expert panel, often called the Pharmacy and Therapeutics Committee (P&T), is a group of pharmacists, physicians, nurses, and administrators as well as laboratory, respiratory, and dietary leaders assembled for the purpose of providing guidance and validation for pharmaceutical utilization in a given organization. I have been a member and chair of many of these committees during my career and will provide you with first-hand evidence of what factors lead to a successful P&T committee and formulary system. The composition and structure of this group can affect greatly both the process, i.e., the formulary system, and the outcome, i.e., the formulary. In my 1982 study of Ohio hospital formularies, although the results suggested that having a formulary assists in the avoidance of ineffective drug products, the effect of process variables on formulary existence or positive outcomes was not discernable [8]. In fact, misconceptions about the formulary expressed by physicians during P&T committee deliberations at one of the largest public hospitals in the midwestern U.S. illustrate the difficulty in forging institutional drug policy [9]. In this report, Rucker and Schiff suggested that the concept of the drug formulary is formed in the minds of many physicians on the basis of negative myths or clinical stories that are inconsistent with its true purpose, i.e., the rational prescribing of drugs of choice. Many of these myths stem from the belief and perception that a formulary interferes with the exercise of individual clinical judgment. This clinical myopia is intensified further by problems found in the literature regarding its purpose and definition as well as distorted evidence from industry, generalizations of anecdotal observations, and the impact of new drug discovery. Other factors that compound objective evaluation include direct and indirect conflicts of interest, such as stock ownership, research support for clinical “seed trials,” speakers’ bureau participation, unrestricted educational grants, and even personal or familial relationships with pharmaceutical sales representatives [10,11].

Moreover, perspectives of the committee members may vary due to the relative intensity of drug therapy in their area of specialization within medical practice, their confidence in drug treatment both as a modality of care in general and as treatments of first choice for particular diagnoses, as well as their views on self-regulation and the role of medical practice in society. However, it seems that a great deal of difficulty may be generated from the inability of members to avoid making generalizations on the basis of anecdotal experiences, or what Rucker called casual empiricism. Casual empiricism is the tendency to categorize on the basis of the extremes of pharmacotherapy effectiveness and safety derived from the treatment of individual patients. Physicians may think that issues of drug selection cannot be separated from the value and outcomes they expect to derive in individual patients [12,13]. In addition, pharmacist and physician members may have differing attitudes regarding medication use in general [14]. As Pierpaoli noted in reference to the rising cost of pharmaceuticals, “A medical staff is successful in influencing the adoption and use of new drug products to the extent that it is a self-disciplined, reasonably cohesive body that is committed to the hospital’s mission to its patients and the community” [15]. Nonetheless, concerns have been raised regarding the lack of comparative drug evaluation and research performed at any level that would provide an expert panel with evidence for making scientifically based drug product selections [16,17,18]. Relative safety and efficacy may be incorporated as public policy in future amendments to the Food, Drug, and Cosmetic Act.

For a drug formulary to work in a given environment, the expert panel must share certain values and a unified mission [19,20,21]. From a social scientific viewpoint, Henriksen identified three major levels of expert panel involvement in drug use in Scandinavian countries: (1) rationalization of the drug assortment; (2) evaluation of consumption and compliance with recommendations; and (3) organization of information services on drug use [22]. Expert panels became prominent because of qualitative and quantitative changes in drug production and marketing strategies employed by industry, the expansion of public health sector medicine, and organized macro-level drug control systems.

## 3. The Environment of Application: The Provider Side

At the hospital or health system level of analysis, a formulary attempts to optimize pharmacological utility for the population of, for example, post-operative bowel surgery patients. It cannot, in itself, perform this function at the individual patient level. A formulary might indicate the optimal dosage and duration of intravenous antibiotic use and equipotent narcotic guidelines based on available scientific literature and local experience. It does not, however, dictate the treatment plan for individual patients. A formulary attempts to delineate the selection of agents with the greatest probability of achieving a successful outcome, which, in this case, is the prevention of surgical site infections (SSI), avoidance of a venous clot in the extremities or lungs, pain control, and anti-emesis. Moreover, for the goal of SSI prophylaxis, the formulary would indicate, in rank order, which antibiotics and combinations are likely to meet this objective.

There are, however, other indications for antibiotic therapy. In the general adult medical/surgical hospital, infections of the respiratory and urinary tracts as well as circulatory and endocrine systems are prevalent. From a formulary perspective, the selection of antibiotics takes into account endemic patterns of infection, the therapeutic range for each possible agent, and the empirical nature of antibiotic prescribing. Agents of choice are then derived by relational comparison and optimization of spectrum or coverage. The clinician then appraises whether the individual characteristics of a patient qualify for an exception to the global selection. If, for example, the patient has a history of smoking, alcohol abuse, or cancer, the global choice may be modified by the addition of rifampin or an aminoglycoside. Patients often have co-morbidities, and the formulary establishes normative expectations for prescribing based on institution-wide indicators; clinical judgment is necessary to address inter-patient variation.

Using the antibiotic selection process example in a health system environment presents another set of unique therapeutic options. In this case, patients are followed throughout the continuum of care, which could include ambulatory, acute, sub-acute, rehabilitation, nursing home, and home care. To avoid therapeutic chaos, selection of antibiotics must include those agents with a variety of dosage forms and indications as well as a lower degree of dose administration complexity. Ideal candidate agents, or drugs of first choice, may include those whose dose and typical duration is unaffected by route of administration or elimination, toxic metabolites, or drug interactions. Thus, antibiotic selection via the formulary mechanism must account for the process of care in the health system.

The integrative nature of formulary management in the health system presents a broader set of care demands than those at the ward or hospital level. What is efficient in one aspect of the patient’s care may not be in another treatment context. Even if the selection of agents meets the clinical goals of therapy based on scientific evidence, because of arbitrarily derived or artificial classes of trade in the U.S., a health system may pay different prices for the same pharmaceutical. Our ideal agent may not be so ideal if, for example, the health system receives free goods of an oral antibiotic in exchange for use of the intravenous form for acute care patients but is charged average wholesale price for its long-term and home care patients. The scenario is complicated further by the scope of pharmaceutical care provided and licensing requirements of pharmacies within the health system by each state.

## 4. The Environment of Application—The Payer Side

Payers view formularies differently than care providers, especially for the treatment of ambulatory patients. Formularies are considered by many as a cornerstone of effective drug therapy benefit management [23]. Formularies in managed care organizations (MCOs) often are administered by pharmacy benefit management companies (PBMs). PBMs work closely with MCOs to structure contracts that integrate subscriber needs and manufacturer rebate/discounting provisions. Many PBMs have P&T committees similar to those found in hospitals and health systems and use a structured technology assessment protocol to approve new products for reimbursement, to develop protocols for responsible utilization (such as generic and therapeutic substitution, gastroesophageal reflux disease (GERD) therapy, or pneumonia treatment), and to delete products through therapeutic class review. Manufacturers typically seek incentive contracts with PBMs in exchange for guarantees of market share movement toward agreed upon targets. Often, a manufacturer with a broad product assortment will link the centerpiece product with those in other categories. For example, depending on the degree of multiplicity and similarity within a pharmacological class, a company that produces a histamine-2 receptor antagonist may bundle their hydroxymethylglutaryl-CoA synthase inhibitor or calcium channel blocker into a contract to increase utilization. In many cases, bundling not only signifies preferred products but also identifies preferred pharmaceutical companies in a relationship that resembles a partnership. In the 1990s, several top pharmaceutical companies, including Merck and Eli Lilly, sought to capitalize on this strength and formalized partnerships by purchasing PBMs for the purpose of moving the market share of their branded products.

Payer-driven formularies actually may work more cost efficiently than provider-based counterparts, although no data exists to verify this supposition. Many formularies specify exclusions by route of administration, therapeutic category, and prescriber type. The patient may be penalized in the form of higher copays or even be required to pay the usual and customary price for non-formulary prescriptions written by unauthorized prescribers. In the past, the prescriber may have received a “Dear Doctor letter” from the carrier, encouraging appropriate choice and utilization of formulary products on behalf of individual patients. However, with the advent of electronic prescribing, prescribers now often receive these notices at the point-of-care in the process of prescription generation. Often, prescribers are contacted directly by dispensing pharmacies to change prescriptions to approved products or to alter the duration of therapy based on protocols of use developed by the PBM.

Unlike provider-based formularies, payer-driven formularies have the difficulty of assessing off-label uses of approved products. To mitigate risk, many PBMs and specialty pharmacy divisions employ explicit use criteria through a prior authorization (PA) mechanism for high cost drugs, i.e., those that cost more than $1000 monthly. In the past, the indication for pharmacotherapy is not part of the adjudication for reimbursement on individual patients. Moreover, the reimbursement system does not address the risk of medication use for pre-existing conditions. PAs for non-formulary drug products or indications is more structured than those found in many institutional formularies. This may be a result, in large part, to time frame and urgency of treatment. Payer-based formularies do not typically authorize the use of injectable drugs (except insulins and other specialized programs like those for interferon, disease-modifying anti-rheumatic drugs (DMARDs), and erythropoetics) or products available without prescription. Because their formularies are designed for ambulatory patients, PBMs usually cover products administered by oral and topical routes. Historically, oral contraceptives and appetite suppressants were usually not part of the benefit; however, many have well-developed use limitations and criteria. Medications prescribed while the patient recovers in a rehabilitation center or nursing home are also excluded in the pharmacy benefit but may be covered under other forms of insurance, such as major medical and gap policy instruments.

## 5. Interprofessional Relationships

Formulary decision-making is not widely understood or studied, even today. It is known that most committees operate via consensus of opinion. Interviews with directors of pharmacies and physician chairpersons of P&T committees, conducted as part of a 1980 study of superior hospital formularies identified several aspects which may elude the casual observer [21]. In these interviews, the operation of a formulary as an instrument of rational drug therapy was dependent on intangible factors like leadership, integrity, and mutual collegial respect. Physician and pharmacist members had developed mature relationships based on the unique and demonstrated contributions each role lent to the therapeutic process. Often, the pharmacist member attributed this success to the physician and vice-versa. Although absent in the literature, trust plays a significant role in the formulation of institutional drug policy. In my experience and research, the leadership of successful committees goes to great lengths to select members with a high level of professional credibility and dependability. Indeed, while physicians often occupy formal leadership roles, such as chairperson, the pharmacist member of the committee may serve as the conscience of the institution regarding pharmacotherapy. The pharmacist may provide the therapeutic fortitude essential in making difficult policy decisions.

Other members of the committee—nurses, administrators, and other professionals like dietitians—are needed to contribute to therapy decisions within the purview of their expertise. The selection of the nursing practice representatives often is attributable to a specific individual’s ability to communicate and affect change at the unit or ward level as well as represent nurse care related to drug administration and monitoring procedures. Formulary changes may require in-service training for the enactment of drug policy decisions, modalities, and specialized patient monitoring. Administrative support typically is needed for cost of therapy considerations and for communication to the medical staff and hospital board of directors. In addition, the administration may deal with contract review and partnership strategies with the pharmaceutical industry or with buying groups. Dietitians make important contributions regarding food service and advanced nutrition support, especially in the care of patients with diabetes, renal impairment, and malabsorption syndromes. Patient advocates, bioethicists, and attorneys, although recommended on the expert panel of a managed care organization (MCO) or health maintenance organization (HMO), are not usually members of hospital-based panels.

It could be argued that, as a clinical instrument, the formulary is predicated on the role pharmacy is perceived to play in actual direct patient care. In the institutional environment, if the practice of clinical pharmacy is unit-based and patient-driven, other professionals are more able to appraise a pharmacist’s unique activities; pharmacists can be repositories of knowledge at both the patient and policy level. Direct involvement and assumption of responsibility for identifying and managing drug-related problems complements sensible product selection policies.

On the payer side, the application of a formulary could have the greatest impact in therapeutic areas with many drug therapy choices (such as dyslipidemias, GERD, depression, and hypertension), a high degree of process variation (such as bronchial pneumonia, sinusitis, and other infectious diseases), high relational or marginal cost, and conditions in which the failure of pharmacotherapy could lead to additional morbidity or mortality (such as venous thromboembolism (VTE) prophylaxis, heart failure, and estrogen-replacement therapies). However, the identification and management of these problems is complicated by the absence of specific reasons or indications for drug therapy. Claims are processed for individual patients without the assignment and collection of pharmacotherapy rationales or diagnoses despite the significant progress made through integrating medical and pharmacy benefit claims databases. At times, payers are limited in their ability to evaluate the impact of various therapeutic inputs or the short- and long-term outcomes of care. Nevertheless, in comparison to hospital or health system formularies, physician involvement and a general level of confidence in a pharmacy’s ability to maintain and enhance the therapeutic relationship are both essential to formulary success. Moreover, the degree to which a pharmacy is perceived as a problem-solving clinical practice may dissipate the intransigence often encountered in formulary development and administration. P&T committees in any environment are wise to communicate policy selection criteria to and actively solicit feedback and inputs from their professional constituents to avert non-compliance with the formulary.

## 6. Formulary Evolution—From Drug Lists to Electronic Prescribing

Traditionally, formularies evolved from applications in institutional environments to third party payers, including governmental and private. Electronic prescribing mechanisms now allow for formulary checks at the point of care, regardless of environment. Providers receive information in real-time about the coverage parameters for formulary and non-formulary items [24]. An excellent and brief description of formulary progress during the twentieth century indicated that drug lists began within the military in the 1940s, and, in the 1950s, a minimum standard for hospitals called for the establishment of a formulary. Upon implementation of Medicare, drug formularies became included in Joint Commission requirements [3]. The current state of formularies has morphed into a comprehensive set of medication-use policies, algorithms, and preferred agents. Formulary decisions for a given population or subscriber list are often informed by cost-benefit analyses, especially for high-cost medications used in specialty pharmacy practice, although just exactly how these cost containment measures are employed around the world is unclear [25].

From the preceding discussion, it is clear that a formulary is much more than a drug list of authorized products. It is the dynamic result of the nature of therapeutic agreements made among physicians, pharmacists, administrators, and others within hospitals and health systems, as well as governmental and private payers. In many cases, a formulary is a kind of contract that is substantiated by medical staff or plan bylaws, implemented through the pharmacy or medication management function, and monitored by quality improvement processes. After all, a formulary purports to improve the general level of prescribing performance and minimize expenses related to drug therapy. In the next lecture, we will discuss a “nuts and bolts” approach to formulary management as well as the vital need for a formulary in the absence of government approval or licensing of drug products.

## References

[1] American Society of Hospital Pharmacists (1991). ASHP technical assistance bulletin on drug formularies. Am. J. Hosp. Pharm..

[2] American Society of Health-System Pharmacists Principles of a Sound Drug Formulary System. https://www.ashp.org/-/media/assets/policy-guidelines/docs/endorsed-documents/endorsed-documents-principles-sound-drug-formulary-system.ashx.

[3] Tyler L.S., Cole S.W., May J.R., Millares M., Valentino M.A., Vermeulen L.C., Wilson A.L., ASHP Expert Panel on Formulary Management (2008). ASHP guidelines on the pharmacy and therapeutics committee and the formulary system. Am. J. Health-Syst. Pharm..

[4] Rucker T.D. (1982). Formularies: Conceptual and experiential factors related to product selection. Drug Inf. J..

[5] Parrish R.H., Berger B.A. (1985). Ohio hospital formularies: An analysis of structure, process, and outcome. Hosp. Pharm..

[6] Barrett C.W., Wilson A.R., Baker J.A., Lawson D.H., Richard R.M.E. (1982). Formularies. Clinical Pharmacy and Hospital Drug Management.

[7] Schondelmyer S.W. (1994). Competition and Pricing Issues in the Pharmaceutical Industry.

[8] Rucker T.D., Schiff G. (1990). Drug formularies: Myths-in-formation. Med. Care.

[9] Talley C.R. (1978). Hospitals defend formulary decisions against industry attacks. Am. J. Hosp. Pharm..

[10] Hoffman R.P. (1984). Perspectives on the hospital formulary. Hosp. Pharm..

[11] Segal R., Hepler C.D. (1982). Prescribers’ beliefs and values as predictors of drug choices. Am. J. Hosp. Pharm..

[12] Hepler C.D., Clyne K.E., Dorta S.T. (1982). Rationales expressed by empiric antibiotic prescribers. Am. J. Hosp. Pharm..

[13] Bootman J.L., Hurd P.D., Gaines J.A. (1982). Physician and pharmacist attitudes toward medication use. Am. J. Hosp. Pharm..

[14] Pierpaoli P.G. (1993). The rising cost of pharmaceuticals: A director of pharmacy’s perspective. Am. J. Hosp. Pharm..

[15] Kelly W.N., Rucker T.D. (1994). Considerations in deciding which drugs should be in a formulary. J. Pharm. Pract..

[16] Rucker T.D. (1980). The top-selling drug products: How good are they?. Am. J. Hosp. Pharm..

[17] Davis N.M. (1983). The foremost problems associated with formulary management. Hosp. Pharm..

[18] Rucker T.D. (1984). Macro/micro strategies for improving hospital formulary performance. Clin. Res. Pract. Drug Regul. Aff..

[19] Rucker T.D. Formulary administration: Some guidelines for improving performance. Proceedings of the Mid-Winter Clinical Conference, Pennsylvania Society of Hospital Pharmacists, University of Pittsburgh.

[20] Zilz D. (1975). Total medical participation in formulary revision. Drug Intell. Clin. Pharm..

[21] Rucker T.D. (1982). Superior hospital formularies: A critical analysis. Hosp. Pharm..

[22] Henriksen H.H. (1982). A social scientific view on the development of pharmacy and therapeutics committees. Soc. Sci. Med..

[23] Covington T.A., Thornton J.L., Ito S.M., Blackburn S. (1985). The formulary system: A cornerstone of drug benefit management. A Pharmacist’s Guide of Principles and Practices of Managed Care Pharmacy.

[24] Crosson J.C., Schueth A.J., Isaacson N., Bell D.S. (2012). Early adopters of electronic prescribing struggle to make meaningful use of formulary checks and medication history documentation. J. Am. Board Fam. Med..

[25] Acosta A., Ciapponi A., Aaserud M., Vietto V., Austvoll-Dahlgren A., Köster J.P., Vacca C., Machado M., Diaz Ayala D.H., Oxman A.D. (2014). Pharmaceutical policies: Effects of reference pricing, other pricing, and purchasing policies. Cochrane Database Syst. Rev..

